# Behavior of Buff-Breasted Sandpipers (*Tryngites subruficollis*) during Migratory Stopover in Agricultural Fields

**DOI:** 10.1371/journal.pone.0008000

**Published:** 2009-11-24

**Authors:** John P. McCarty, Joel G. Jorgensen, L. LaReesa Wolfenbarger

**Affiliations:** 1 Department of Biology, University of Nebraska at Omaha, Omaha, Nebraska, United States of America; 2 Nongame Bird Program, Nebraska Game and Parks Commission, Lincoln, Nebraska, United States of America; Stockholm University, Sweden

## Abstract

**Background:**

Understanding the behavior of birds in agricultural habitats can be the first step in evaluating the conservation implications of birds' use of landscapes shaped by modern agriculture. The existence and magnitude of risk from agricultural practices and the quality of resources agricultural lands provide will be determined largely by how birds use these habitats. Buff-breasted Sandpipers (*Tryngites subruficollis*) are a species of conservation concern. During spring migration large numbers of Buff-breasted Sandpipers stopover in row crop fields in the Rainwater Basin region of Nebraska. We used behavioral observations as a first step in evaluating how Buff-breasted Sandpipers use crop fields during migratory stopover.

**Methodology/Principal Findings:**

We measured behavior during migratory stopover using scan and focal individual sampling to determine how birds were using crop fields. Foraging was the most frequent behavior observed, but the intensity of foraging changed over the course of the day with a distinct mid-day low point. Relative to other migrating shorebirds, Buff-breasted Sandpipers spent a significant proportion of their time in social interactions including courtship displays.

**Conclusions/Significance:**

Our results show that the primary use of upland agricultural fields by migrating Buff-breasted Sandpipers is foraging while wetlands are used for maintenance and resting. The importance of foraging in row crop fields suggests that both the quality of food resources available in fields and the possible risks from dietary exposure to agricultural chemicals will be important to consider when developing conservation plans for Buff-breasted Sandpipers migrating through the Great Plains.

## Introduction

Row-crop agriculture is one of the most intensive human uses of land. Many bird species use these heavily modified landscapes [1] and both positive [2], [3] and negative [4], [5] effects on birds have been documented. The implications for bird populations using agricultural lands as habitat are not always clear. In some cases agricultural land may provide a suitable substitute for native habitats, while in other cases birds may be forced to use agricultural lands that are not suitable because other, better habitats are unavailable. The first step in identifying possible resource needs is to document what behaviors birds are engaged in while using the habitat type. Likewise, the first step in evaluating risks is to document possible modes of exposure to risk, such as ingesting contaminants while foraging or while preening, or by dermal exposure during bathing.

Many shorebirds use agricultural fields during part of their annual cycle [6]–[8], and several of these species are of conservation concern, including the American Golden Plover (*Pluvialis dominica*), Mountain Plover (*Charadrius montanus*), Long-billed Curlew (*Numenius americanus*) and Buff-breasted Sandpiper (*Tryngites subruficollis*) [9], [10].

Buff-breasted Sandpipers are listed as “highly-imperiled” in the U.S. Shorebird Conservation Plan [9], [10]. They are found in agricultural fields during migration and on their wintering grounds in South America [11]–[13]. During their spring migration, a large proportion of the world's population of Buff-breasted Sandpipers stops in corn and soybean fields in the eastern Rainwater Basin region of Nebraska [14]. While it appears that individual birds spend no more than a few days in Rainwater Basin (Joel Jorgensen et al. unpublished data), the concentration of such a large portion of an at-risk species in agricultural fields raises concerns about how this use of a human-dominated landscape might impact the population.

Buff-breasted Sandpiper use of agricultural habitats raises concerns both about whether the quality of habitat is sufficient to meet the needs of the birds and about the risk posed by exposure to agricultural chemicals while in fields. One step in evaluating the resources used by Buff-breasted Sandpipers and their exposure to hazards associated with agriculture is to determine what behaviors individuals exhibit while occurring in row-crop habitats. In particular, the extent to which birds forage in dry upland fields and in wetlands and flooded fields was not known. While migratory stopover is often associated with intense foraging, Buff-breasted Sandpipers captured during stopover had significant fat reserves (Joel Jorgensen et al. unpublished data), suggesting that the Rainwater Basin may be primarily a resting or staging area.

We studied the behavior of Buff-breasted Sandpipers in agricultural fields during their spring migratory stopover in the Rainwater Basin region of south central Nebraska. We measured behavior of individuals and groups to determine how they were using resources during stopover. We also evaluated how behaviors might change during the course of a day and through the season.

## Results

We conducted 170 flock scan samples. Observations occurred at 75 different locations on 24 different days. Mean number of birds included in the flock scan was 11.5±0.9 birds (all means given as ±1 SE, range = 1 to 95). Approximately half of all birds observed during scans were engaged in foraging behavior (Table 1). Birds resting, engaged in maintenance behavior, social interactions, and walking accounted for between 8 and 16% of observations, while less than 3% of individuals were classified as alert (Table 1).

**Table 1:** Time budgets for Buff-breasted Sandpipers in agricultural habitats.

		Foraging	Social Interactions	Walking	Maintenance	Resting	Alert
Flock Scan	% of birds	51.0±3.6	8.2±1.3	12.3±2.0	10.2±2.0	15.9±2.3	2.4±0.7
Focal Animal	% of time	48.4±4.1	5.7±1.4	12.2±2.3	12.5±2.5	14.9±2.8	6.2±1.4

For Flock Scans, the percent of birds engaged in each behavior category is given. For Focal Animal observations, values represent the percent of the total time engaged in each behavior. Values are given as mean ±1 SE.

To compare behaviors observed during scan samples in different habitat types, we classified wetland sites, including temporarily flooded agricultural fields, as “wet” and agricultural fields without standing water as “upland”. We only observed flocks in “wet” areas after 12:00 h and we restricted the analysis to those scans conducted after 12:00 h. Behaviors included in this comparison were foraging, maintenance, resting, and walking. Social interactions and alert behaviors were not observed in wetlands frequently enough to include in our analyses. The behaviors of individuals in flock scans were significantly different between the two habitat types (Table 2; Wilks' Lambda = 0.73; *F*_4, 49_ = 4.4, P<0.004). A higher percentage of birds was observed foraging at upland agricultural fields without standing water (51.0±4.4%) than at wetlands and agricultural fields with standing water (21.3±10.0%; *F*_1,52_ = 7.5, *P*<0.009). Significantly fewer birds were engaged in maintenance behavior in upland fields (6.9±2.4%) than in wet sites (27.4±5.4%; *F*_1,52_ = 12.3, *P*<0.001). Differences between habitat types for the two other prevalent behavior categories (resting and walking) were not significantly different (Table 2; *P*-values>0.10).

**Table 2:** Behavior of Buff-breasted Sandpipers in dry upland sites differed from behavior at wetlands.

		Foraging	Walking	Maintenance	Resting
Upland	% of birds	51.0±4.4	12.5±2.4	6.9±2.4	18.9±3.7
Wetland	% of birds	21.3±10.0	7.4±5.4	27.4±5.4	30.7±8.4
		F1, 52 = 7.5	F1, 52 = 0.75	F1, 52 = 12.3	F1, 52 = 1.70
		P = 0.009	P = 0.40	P<0.001	P = 0.20

Behavior profiles give the percent of birds engaged in each behavior category based on Flock Scans. Flocks of Buff-breasted Sandpiper were only observed at wetlands in the afternoons and evenings so the behavior profile of birds in dry uplands includes only observations obtained after 1200 h. Overall behaviors differ between habitat types (Wilks' Lambda = 0.73; *F*_4, 49_ = 4.4, P<0.004); *F* and *P* values in the table show the results of univariate tests of differences between habitats for each behavior category. Values are given as mean ±1 SE.

We limited subsequent analyses of foraging behavior to the data collected at upland sites. We conducted scan samples in 58 upland locations. Scan samples were collected between 2 and 20 May. The percent of individuals in groups observed foraging increased over the course of the season (b = 0.034; *F*_1, 54_ = 9.0, *P*<0.004).

We conducted 125 focal animal samples. Observations occurred at 65 different locations on 20 days. Focal birds spent approximately half their time foraging (Table 1). Walking, maintenance, and resting accounted for 12 to 15% of the time of focal individuals while they spent less than 10% of their time in social interactions and being alert (Table 1).

Focal animal observations were conducted from 2 to 19 May. The percent of time focal individuals spent foraging showed an increase over the course of the season (b = 0.03; *F*_1, 61_ = 9.3, *P*<0.004).

**Figure 1:**
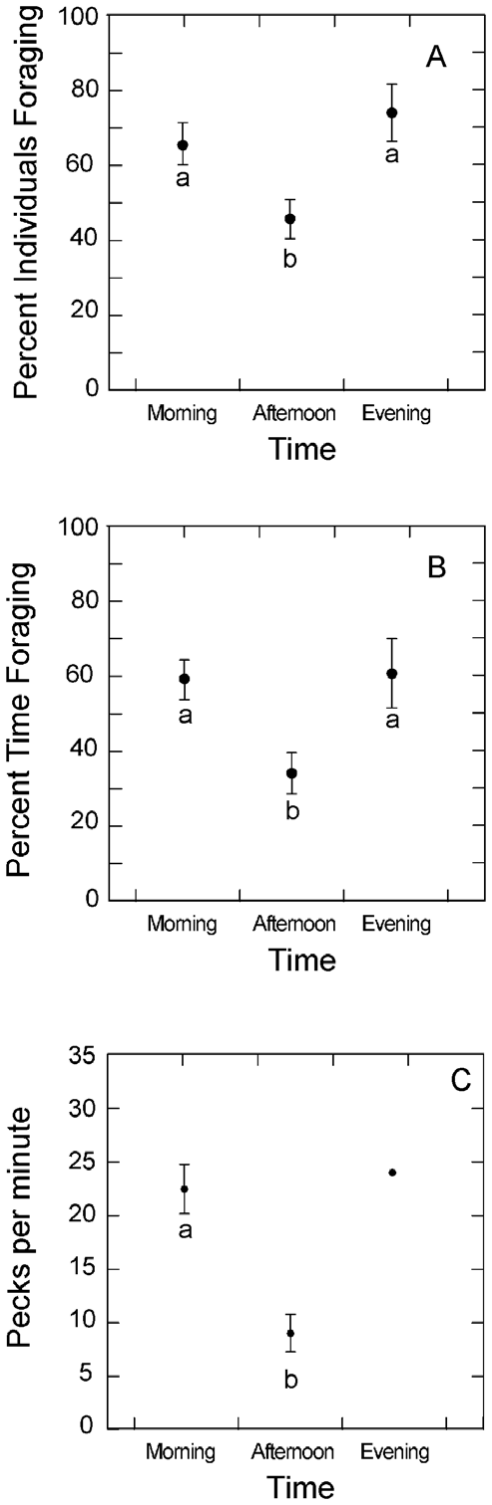
Foraging activity of Buff-breasted Sandpipers changed with the time of day. The mean (± SE) percent of individuals observed foraging during flock scan samples (Fig. 1A) and the percent of time individuals foraged during focal observations (Fig. 1B) were lower during the afternoon (from 1200 to 1800 h) than in the morning or evening (0600 to 1200 h and 1800 to 2200 h). The intensity of feeding, as measured by the number of pecks per minute while foraging (Fig. 1C), was significantly lower in the afternoon than in the morning (*t* = 4.46, df = 7, *P* = 0.0043; only one observation was available during the evening time period). Means that share a letter are not significantly different using a post-hoc Tukey PLSD test.

Both scan samples and focal samples detected changes in foraging behavior over the course of a day. The amount of time devoted to foraging declined during mid-day for both sets of samples (Scan sample Fig. 1A, *F*_2, 54_ = 5.0, *P* = 0.009; Focal individual sample Fig. 1B, *F*_5, 50_  = 3.69, *P*<0.006).

We collected information on foraging rates during 30 observations at 8 locations. The mean foraging intensity was 12.1±1.5 pecks per minute (range = 2 to 34 pecks per minute) and was significantly higher in the morning than in the afternoon (Fig. 1C, *t* = 4.46, df = 7, *P* = 0.0043).

## Discussion

Foraging was a primary activity for Buff-breasted Sandpipers during stopover in agricultural fields in the Rainwater Basin. Comparatively little foraging was observed when birds were at wetlands. Instead wetlands were visited for short periods of time and used for bathing and drinking. While foraging was the most common activity, it was not as prevalent as observed in some other shorebirds during migratory stopover [15], [16]. For example, in the southern Great Plains shorebirds such as Least Sandpipers (*Calidris minutilla*), Western Sandpiper (*Calidris mauri*), and Long-billed Dowitchers (*Limnodromus scolopaceus*) spent over 70% of their time foraging [17]. Within the Rainwater Basin region, shorebirds associated with wetlands varied in what proportion of their time was spent foraging, though overall foraging intensity was similar to what we observed by Buff-breasted Sandpipers in upland agricultural sites [18].

Our observations were restricted to daylight hours. Some shorebirds feed actively at night and the patterns of activity can vary significantly between day and night [16], [19]–[21]. While we did not quantify behavior of Buff-breasted Sandpipers at night, we did have the opportunity to observe them in agricultural fields after dark. During this same time period we spotlighted and captured birds at night generally from 1 hr after sunset to 03:00 h. We did not observe evidence of foraging after dark. Instead, birds were observed resting and sleeping in agricultural fields at night. There can be important differences in how shorebirds are using agricultural fields during the day and night [22]. Because Buff-breasted Sandpipers are using the fields at night, additional details about their behavior at night will contribute to a better understanding of their habitat needs during stopover.

Buff-breasted Sandpipers spent between 5% and 8% of their time in social interactions, including both courtship and aggressive interactions (Table 1). This amount of interaction during migratory stopover is high relative to other studies of shorebirds where courtship is rarely reported and aggression typically involves less than 1% of species' time budgets [15], [17]. We did not attempt to separate aggression and courtship behaviors in our analyses since most cases where social behavior was observed involved a rapidly changing mix of single wing flashes, double wing courtship displays, and chases [23], [24]. The relatively high intensity of social interactions displayed by Buff-breasted Sandpipers compared to other shorebirds raises the possibility that spring migratory stopover in the Rainwater Basin may play an important role in group or pair formation [24].

The daily patterns of feeding by birds in agricultural habitats can be an important variable in evaluating the risk from pesticide exposure. A recent U.S. Environmental Protection Agency Scientific Advisory Panel noted that information about the temporal pattern of feeding was a key variable for understanding exposure to and risk from pesticides, especially pesticides that are rapidly metabolized such as carbofuran [25]. In current risk models, the risk from dietary pesticide exposure is much lower when the daily intake of food is spread over the course of an entire day than it is when feeding is concentrated in the morning and evenings. For most bird species, there is not sufficient information about feeding patterns of adults in agricultural fields to parameterize risk assessment models [25]. Our results indicate that foraging behavior may not be evenly spread over the course of day and that it is not reasonable for risk models to assume that food intake occurs at a steady rate over the course of a day. Feeding patterns are likely to be species- and habitat-specific. For example, in non-agricultural wetlands of the Great Plains some shorebirds show daily cycles of feeding activity while other species feed at a steady rate through the day [15], [17].

We do not know if agricultural chemicals pose a risk to Buff-breasted Sandpipers and in general the magnitude of risk to birds posed by currently used agricultural chemicals is difficult to quantify. Restrictions on the use of some highly toxic chemicals such as carbofuran in the United States has almost certainly reduced the level of acute risk to birds [26], although these chemicals may still be used in other countries and their continued use in the United States is still a subject of debate [25]. The switch to chemicals with lower persistence in the environment reduces avian risk but it has also increased the already difficult challenges of quantifying mortality [27], [28] or sublethal effects [29], [30], and has increased the reliance on exposure and risk models [31], [32]. An understanding of how birds interact with their environment is critical to developing robust models of avian exposure and risk.

The importance of foraging as the main behavior during migratory stopover by Buff-breasted Sandpipers indicates that both the quality of foraging habitat provided by agricultural fields and possible dietary exposure to agricultural chemicals should be evaluated. The intensive use of agricultural fields during migratory stopover in the Rainwater Basin and other agricultural landscapes in the Great Plains by Buff-breasted Sandpipers and other at-risk species emphasizes the need to incorporate a broad array of habitats as part of shorebird conservation planning [33].

## Materials and Methods

Buff-breasted Sandpipers were observed during migration through the eastern Rainwater Basin region of Nebraska in May of 2007 and 2008. This distinct geologic region is dominated by corn and soybean agriculture and contains numerous playa wetlands (for detailed descriptions of the study area see [8], [34]).

Individuals and groups of birds were located by scanning crop fields and wetlands throughout the daylight period. While Buff-breasted Sandpipers did make short visits to wetlands for bathing and drinking, the vast majority of their time was spent in agricultural fields. During spring migration these crop fields were bare, with only scattered stubble remaining from the previous years crops. By the end of spring migration some fields contained newly emerged corn plants (<10 cm tall).

When Buff-breasted Sandpipers were located, we used scan samples of groups of birds separated by focal animal observations to quantify behavior [35]. Scan samples and focal animal observations should each be effective for providing estimates of time budgets and combining the methods should help balance their relative strengths and weaknesses [36]. For scan samples the field containing Buff-breasted Sandpipers was systematically scanned using a spotting scope for approximately one to three minutes. During the scan the behavior of each individual when it was first spotted was recorded. Scans proceeded systematically from one side of the field to the other to reduce bias resulting from more conspicuous behaviors being more likely to be observed.

In crop fields, we followed scan samples with focal animal samples whenever birds remained long enough to complete a focal observation. In wetlands, only scan samples were conducted. For focal observations one individual bird was selected at random. This was done by selecting a random number between 1 and *n*, where *n* was the number of birds detected during the flock scan sample. The field was then systematically scanned until the *n*th bird was located. This bird became the focal individual for a 3-minute focal observation. Birds were observed using a spotting scope, and each time behaviors changed the time was recorded to the nearest second. If the focal bird disappeared from sight before the observation period was half over the observation ended and the data were discarded. If the observation period was more than half completed when the focal bird was lost from sight, the time was recorded and the data were included in the analysis, correcting for the length of the observation period.

Behaviors recorded included: 1) foraging (defined as birds pecking at the ground); 2) social interactions including courtship, chasing, and wing flash displays); 3) walking (locomotion not involving foraging or social interactions); 4) maintenance behaviors (including preening, dust bathing, and water bathing); 5) resting (defined as sleeping or standing); and 6) alert (birds stationary with neck upright or head tilted to look at the sky).

In 2008, a subset of focal animal observations were followed by observations of foraging intensity. After the 3-minute focal observation was completed, we recorded the number of foraging pecks by the focal individual over a 1-minute period while they were actively foraging. Each distinct jab or peck at the foraging substrate was counted. Observations began as soon as the focal bird began foraging. If the focal individual stopped foraging or was lost from sight before 30 seconds elapsed the data were discarded: if foraging was observed for between 30 and 60 seconds the data were included in the analyses, correcting for the length of time the individual foraged. In three cases observations lasted longer than 60 seconds: these observations are included in the analyses, correcting for the length of the observation period.

To evaluate changes in behavior over the course of a day, we divided the day into three time periods (morning: 06:00 to 12:00 h, afternoon: 12:00 to 18:00 h and evening: 18:00 to 22:00 h).

We used multivariate analysis of variance to determine whether significant differences in the number of individuals engaged in foraging, walking, maintenance and resting occurred between “wet” areas and upland areas. Our data met assumptions of no outliers, homogeneity within the variance-covariance matrices (values were all within a factor of ten of each other), and the absence of multicollinearity of explanatory variables [37]. Because the number of scans or focal observations per site ranged from 1 to 8, we averaged data collected for each location. We did not conduct observation in the same location on different days. For six locations, observations spanned more than one time category, and we randomly selected one category to include in the analysis. Effects of time of day and date on foraging were evaluated using the general linear model function of JMP version 5 and specifying time of day and date as fixed effects. We used Tukey PLSD tests for post-hoc comparisons of means for time categories.
